# Clinical Trials Cannot Substitute for Health System Strengthening Initiatives or Specifically Designed Health Policy and Systems Research

**DOI:** 10.1080/15265161.2016.1170242

**Published:** 2016-06-01

**Authors:** Kwaku Poku Asante, Caroline Jones, Sodiomon Bienvenu Sirima, Sassy Molyneux

**Affiliations:** Kintampo Health Research Centre; University of Oxford and KEMRI Wellcome Trust Research Programme; Centre National de Recherch et de Formationsur le Paludisme; University of Oxford and KEMRI Wellcome Trust Research Programme

Denburg and colleagues (2016) present an interesting hypothesis that the existence of clinical trials infrastructure may function as a quality improvement lever for all patients in an institution or region, independent of their individual participation in trials. They argue that this hypothesis, if correct, justifies enhanced research capacity in low- and middle-income countries (LMICs) as a pillar of health systems development.

We draw on our wide experience of running and studying clinical trials (CTs) in Sub-Saharan Africa, and of conducting health systems and research ethics studies, to provide illustrative examples and give support to several points raised by Denburg and colleagues. We only consider CTs that aim to generate social value in the countries in which the research is conducted (Emanuel, Wendler, and Killen 2004), recognizing that this is far from the case for some trials conducted in the region (Petryna 2007). In particular, we draw on a mixed-methodology study we conducted across CT sites in Kenya, Burkina Faso, and Ghana (Angwenyi, Asante, and Traore 2015): a study aimed at understanding the implications of what are often substantial and diverse CT inputs for the health services and systems in which they are embedded. Funded by the European Developing Country Trial Partnership, the study included health facility audits as well as in-depth interviews and questionnaires with health workers and managers and CT staff (Angwenyi et al. 2015).

Overall, we agree with Denburg and colleagues that CTs can have positive effects on the health services and systems in which they are embedded, and that these effects should be included in the appraisal of trial benefits. We organize our reflections on these positive effects using the World Health Organization (WHO) health systems building blocks framework, given its familiarity to many. Illustrative examples—beyond the intended social value of the findings, and research capacity building—include:

- Health service delivery: New or strengthened services run by CT funded staff (e.g., a tuberculosis [TB] or sickle-cell clinic) reportedly alleviate workload in existing services. Where accessible to everybody as opposed to study participants only, many people potentially benefit, and facility resources may be released for other health system needs (Tinto, Valea, and Sorgho 2014; Njue, Kombe, and Mwalukore 2014).
- Human resources: Clinical and other CT employed specialists—such as pediatricians, pharmacists, health educators, or information technology staff—are said to have provided new inspiration and support to public-sector staff. As part of the setup of trials, existing staff may receive additional income or training that strengthens their motivation and performance, potentially benefiting all facility users (Molyneux, Njue, and Boga 2013).
- Health system information: CTs often require significant data collection for planning purposes (e.g., to calculate trial sample sizes), or as part of monitoring trial implementation and outcome. Where appropriately planned, packaged, and fed back, these data have the potential to be valuable to health managers and providers in supporting diverse services.
- Medical products and technology: There is often the provision of quality controlled medical products that would not otherwise be present in facilities (e.g., new generation drugs, digital x-rays, and ultrasound). Where accessed by nontrial participants, these products can impact positively on care quality (Liheluka, Lusingu, and Manongi 2013).
- Health system financing: We found examples of CT funds being used to pay for participants’ hospital fees or health insurance premiums, and of facilities charging some indirect costs to CTs. Such income has the potential to keep relatively resource-deprived facilities open and running. The high level of financial accountability required by trial sponsors may also strengthen financial accountability, and therefore resources available, in facilities.
- Leadership and governance: CTs establish trial management teams that often include investigators, clinicians, laboratory technologists, and local health authorities. Teams meet regularly to monitor progress using established international guidelines and adherence to local regulations. These interactions may strengthen local leadership skills, strengthen local guidelines, and facilitate the setting up and running of current and future trials in ways that improve service delivery.

Denburg and colleagues note that a limitation of their hypothesis is the potential for a negative trial infrastructure effect. We agree, and would give even greater emphasis to this point, and suggest that consideration of these wider negative effects be included alongside benefits in trial appraisals. We identified many negative impacts of CTs for the systems in which they are embedded, with examples cross-cutting the WHO building blocks including:

- Introducing inequities between participants and non-participants. The benefits to those enrolled in research are a key concern in CT ethics guidelines, and typically described in research protocols. Often not included are details on where those services will be provided and by whom, and how these services will differ for nonparticipants. Where participants and nonparticipants appear to get very different levels of care, significant tensions can be introduced, with unclear implications for family and community relations in the medium and longer term (Molyneux, Njue, and Boga 2013).
- Increasing inequities between participating and nonparticipating services/facilities/regions. More broadly, facilities and regions able to host CTs may already have stronger infrastructures than others, and continue to draw in local resources and improve quality of care, with important equity implications.
- Adding to existing staff workload. Health facility staff members may feel they are being asked to conduct CT activities beyond their job description. Where these roles are not financially supported, their motivation to deliver quality care within the wider health system may be affected. Where staff members are paid a top-up, a precedent may be set for all “additional” activities, including those perceived by health managers to be core health worker tasks.
- Tensions between trial and nontrial staff. Where there are differences between staff members in salary and support levels, or in terms of who is paid top-ups or not, tensions can emerge. In typically very hierarchical health systems, staff may feel unable to raise concerns with managers, with negative implications for motivation and delivery of quality care.
- Realignment of structures and systems to the sponsor rather than provider and patient needs. The structures and systems required by sponsors are necessarily focused on ensuring that the CT is successfully completed, as opposed to ensuring that quality of care for all facility users is maintained.
- Sustainability of CT inputs and benefits: CTs are typically time-bound and conducted over short durations. The sustainability of positive impacts on health services and the broader system when the CTs end is unclear.

To harness some of the positive effects of CTs for health services and systems, and minimize important concerns, we strongly support Denburg and colleagues that there is huge potential in LMICs to develop learning health care systems. Indeed, several of us are actively involved in pursuing this agenda. Nevertheless, these initiatives are ambitious, will take time to evolve, and have their own ethical dilemmas. In the meantime, and in line with many ethics recommendations and guidelines (Emanuel, Wendler, and Killen 2004), we support the need for collaborative partnerships from the outset between CT researchers and health managers (and others), who carefully consider whether and how CTs are embedded within health services and systems, including:

- Where are inputs placed physically? Are they in a shared space? Are they visible to nontrial health service providers and users?
- Who has access to inputs? Can they only be used by CT participants or are there mechanisms to facilitate wider access, and what are the ethical considerations in widening the CT benefits to non-CT participants?
- How do CT inputs link to existing services or staff? Are there efforts to build upon existing services and staff, or are separate and parallel structures and systems set up?
- What happens at the trial conclusion? What plans are in place? Will inputs be handed over to and maintained by the Ministry of Health or nongovernmental organizations (NGOs), or only sustained through further trials? Who will be responsible and for how long?

Responses to these questions are far from straightforward. In contexts of significant resource needs, where do the responsibilities of researchers (and funders) end? Stakeholders have to consider the practical and financial costs involved in broadening access to CT inputs: Improving the collateral benefits of CTs should not come at the expense of being able to conduct locally important— and ideally locally driven—research in LMICs; requiring quality-of-care gains for all patients risks the findings from those trials not being representative to less well-resourced settings, potentially undermining the social value of those trials. Also, efforts to build up and work with the existing health system should not be so overwhelming that ultimately facilities are undermined, especially in the longer term when sustainability concerns are likely to emerge.

These latter points link to the importance of ensuring, as Denburg and colleagues note, that any focus on CTs does not replace basic health system strengthening. We also agree that considering enhanced research capacity as a pillar of health systems development should not undermine that broader goal. We would add that research capacity should include the ability to conduct excellent quality health policy and systems research (HPSR). HPSR focuses on: the performance of health systems and their subcomponents (resources, organizations, and services); consideration of how links among the subcomponents shape performance; and how to strengthen health system performance over time (Sheikh, George, and Gilson, 2014; Senkubuge, Modisenyane, and Bishaw, 2014). HPSR is increasingly recognized as critical to making a difference to public health globally.
